# Adherence to General Diabetes and Foot Care Processes, with Prompt Referral, Are Associated with Amputation-Free Survival in People with Type 2 Diabetes and Foot Ulcers: A Scottish National Registry Analysis

**DOI:** 10.1155/2022/7414258

**Published:** 2022-06-14

**Authors:** Bernardo Meza-Torres, Scott G. Cunningham, Christian Heiss, Mark Joy, Michael Feher, Graham P. Leese, Simon de Lusignan, Fabrizio Carinci

**Affiliations:** ^1^Department of Clinical and Experimental Medicine, University of Surrey, UK; ^2^Nuffield Department of Primary Care Health Sciences, University of Oxford, UK; ^3^Division of Population Health and Genomics, University of Dundee, Scotland, UK; ^4^Surrey and Sussex Healthcare NHS Trust, East Surrey Hospital, Redhill, UK; ^5^Department of Statistical Sciences, University of Bologna, Italy

## Abstract

**Aims:**

To compare different packages of care across care providers in Scotland on foot-related outcomes.

**Methods:**

A retrospective cohort study with primary and secondary care electronic health records from the Scottish Diabetes Registry, including 6,845 people with type 2 diabetes and a first foot ulcer occurring between 2013 and 2017. We assessed the association between exposure to care processes and major lower extremity amputation and death. Proportional hazards were used for time-to-event univariate and multivariate analyses, adjusting for case-mix characteristics and care processes. Results were expressed in terms of hazard ratios with 95% confidence intervals.

**Results:**

2,243 (32.7%) subjects had a major amputation or death. Exposure to all nine care processes at all ages (HR = 0.63; 95% CI: 0.58-0.69; *p* < .001) and higher foot care attendance in people aged >70 years (HR = 0.88; 0.78-0.99; *p* = .03) were associated with longer major amputation-free survival. Waiting time ≥ 12 weeks between ulceration and clinic attendance was associated with worse outcomes (HR = 1.59; 1.37-1.84; *p* < .001). In people > 70 years, minor amputations were associated with improved major amputation-free survival (HR = 0.69; 0.52-0.92; *p* = .01).

**Conclusions:**

Strict adherence to a standardised package of general diabetes care before foot ulceration, timely foot care after ulceration, and specific treatment pathways were associated with longer major amputation-free survival among a large cohort of people with type 2 diabetes in Scotland, with a larger impact on older age groups.

## 1. Introduction

Diabetic foot ulcers (DFUs) are an important and costly complication [1], affecting nearly 2.2% of the people with type 2 diabetes annually [2]. People with DFU are at a greater risk of major lower extremity amputations (LEAs) and have a 40% 5-year mortality rate [3].

Monitoring healthcare providers' outcomes and value-based payment schemes improve quality and control costs in the care of patients with type 2 diabetes and DFU, as widely promoted by the International Consortium for Health Outcomes Measurement (ICHOM) [4]. Process indicators allow monitoring adherence to guidelines in type 2 diabetes across different settings, through the continuous use of reliable measures [5]. For example, an increased level of adherence to individual process measures, e.g., HbA1c and glucose monitoring, is associated with better intermediate outcomes and may prevent cardiovascular and foot complications [6–9]. In the United Kingdom, specific packages of multiple process measures, e.g., the nine care processes, have been developed for the personalised care of people with type 2 diabetes in primary care settings [10, 11]. However, to the best of our knowledge, the impact of the delivery of these packages of care processes for DFU as an index condition and LEA or death as outcomes has not been investigated. This is a relevant research gap, given that people with DFU require specific attention to monitor their prognosis [12].

Access to specific organizational arrangements, e.g., multidisciplinary teams, may decrease the risk of LEA among subjects with type 2 diabetes, once a DFU is diagnosed [13]. Vascular surgery is one key element for the management of critical ischaemia. Podiatry and orthopaedics are also key to inspecting suspicious lesions, treating neuropathic foot infections, managing plantar pressure, and performing minor LEAs (amputations below the ankle) [14]. These specialized therapeutic interventions include the aim of preventing major LEAs (amputations above the ankle) and death. Outpatient visits to lower extremity specialists, cardiovascular specialist, and foot inspection at primary care are process measures currently indicated in clinical guidelines [15]. The effect of timely access to these specialty services after a DFU diagnosis has not been thoroughly investigated from a primary care perspective away from specialist foot clinics, although it has been reported with paucity in national data [16].

Although another study has reported on the progression of DFU [17], the use of longitudinal cohorts extracted from population-based diabetes registries has not been attempted. Until now, the heterogeneity of data sources has hampered the application of standardised definitions to the analysis of major amputations in type 2 diabetes at a global level [18, 19].

In this study, we aimed to use the population-based Scottish Diabetes Registry (SCI-DC) [20] to answer the following research question: Which standards of care are associated with higher rates of major amputation-free survival times in people with type 2 diabetes and foot ulcers adjusting for relevant sociodemographic and clinical covariates, according to routinely collected data?

## 2. Material and Methods

### 2.1. Data Sources

We used data from SCI-DC, a clinically-led framework created in 2002 to improve care in diabetes, including data from all nationwide primary and secondary care units in Scotland [20]. The system feeds national reports and scientific publications extensively used for quality of care monitoring in type 2 diabetes [21, 22]. Data access was approved with the scope of conducting collaborative research between the HEALTHPROS Consortium [23] and the Scottish Diabetes Research Network (SDRN) [24]. Ethical clearance was obtained from the NHS Research Ethic Committee (REC) on September 2019. Research on General Data Protection Regulation (GDPR) and confidentiality training was completed prior to accessing the data safe haven. Anonymized electronic health records were accessible for research purposes following approvals through the SDRN Epidemiology Group: Scottish Government Public Benefit and Privacy Panel for Health and Social Care Approval: 1617-0147.

### 2.2. Study Design and Population

We performed a time-to-event analysis using a retrospective cohort design. The study population was extracted from patient level data in the SCI-DC registry in August 2020, applying the following criteria: (1) including people over 18 years of age diagnosed with type 2 diabetes at 1^st^ January 2013 (start of the study); (2) followed up until 31^st^ December 2017, with a first active foot ulcer recorded between 1^st^ January 2013-31^st^ December 2017 as an index condition; and (3) excluding people with LEA recorded prior to the first recorded active ulcer.

Outcomes were major lower extremity amputation or death. Major LEA was investigated independently. Major LEA and death together were investigated as a combined outcome, which is commonly referred in the scientific literature as “amputation-free survival” [17, 25].

Optimal processes of care received by patients were considered as the main exposure of interest.

### 2.3. Data Definitions

Data definitions were based on the ICHOM Standard Set for Diabetes [26, 27]. It specifies 84 variables, including clinical processes and case-mix characteristics, to be routinely recorded in baseline or spaced intervals to ensure adequate care standards. We mapped the variables in the SCI-DC registry to their equivalent ontological concept in the ICHOM guide for identification and homogenization. Process measures were defined using the SIGN guidelines [15], the Scottish Diabetes Survey [11], the ICHOM and NICE recommendations on care standards [10, 27, 28], and scientific reports [5, 16]. Overlaps between definitions were resolved prior to the extraction of all key columns from the SCI-DC registry.

### 2.4. Data Extraction

LEA and vital status (date of death) were extracted as outcomes for all included patients. Censored observations included patients lost to follow-up prior to 31^st^ December 2017 or without events at that date.

Case-mix variables considered as potential predictors, included lifestyle, demographic, clinical, and comorbidity characteristics, measured according to the ICHOM definitions at the date closest to baseline.

Age and gender were used as the main demographic characteristics, along with a history of 11-comorbid conditions: deprivation score, smoking status, body mass index (BMI), estimated glomerular filtration rate (eGFR), total cholesterol, peripheral vascular disease, claudication, acute myocardial infarction (AMI), cerebrovascular event (CVD), hypertension treatment, and retinopathy. All characteristics were recorded as part of routine clinical practice in Scotland. Deprivation was obtained from the Scottish Index of Multiple Deprivation (SIMD) score as recorded at baseline [29]. Patients were stratified into the most deprived quintiles (SIMD < 3) and the least deprived quintiles (SIMD ≥ 4) for comparisons.

The history of comorbid conditions was obtained from the diagnostic records in the SCI-DC registry. Diagnoses were independently recorded by clinicians during consultations. We did not develop ad hoc diagnostic criteria at the data extraction level.

The following process measures were considered for their relevance to outpatient care: the nine care processes in the six months prior to DFU, the number of foot care visits in the six months prior to DFU, the waiting time between a diagnosis of DFU and foot specialist care, cardiovascular specialty visits after DFU, cardiovascular quality of care score after DFU, and a minor lower extremity amputation after DFU.

Process measures were defined as follows:

- *The Nine Care Processes*. It is defined based on the algorithm used in the annual report of the Scottish Diabetes Survey, according to the best standard of care for people with type 2 diabetes defined by the SIGN guidelines, consistently with the ICHOM and NICE recommendations. They involve the monitoring of HbA1c, BMI, blood pressure, smoking status, retinopathy screening, urinary albumin test, creatinine, total cholesterol, and previous foot risk. Patients were stratified in two categories: receiving all or less than 9 processes during the six months prior to the first DFU
- *Foot Care Visits before DFU*. We calculated the annual rate of visits per patient from the time of first ulceration to the end of study to lower extremity specialists. This included outpatient visits to podiatry, vascular surgery, or orthopaedic outpatient specialist services
- *Cardiovascular Visits after DFU*. We calculated the annual rate of visits per patient from the time of first ulceration to the end of study to cardiovascular specialists. This included outpatient visits to cardiology, cardiovascular surgery, cardiothoracic surgery, or diabetology/endocrinology
- *Foot Care Checks after DFU*. We calculated the rate of the five foot-checks received annually in outpatient care per patient from the time of first ulceration to the end of study. This included the following: monofilament test, pulses, vibration, structural abnormalities, and foot risk scoring, as according to the SIGN guidelines
- *Waiting Time*. We calculated the time from DFU diagnosis to the first appointment with a specialist in the categories ii and iii above. Values were categorized as less or equal greater than 12 weeks to specialist attention. The 12-week cut-off date was defined as a pragmatic time duration for booking routine clinical follow-up considering time-tabling complexities. It is aimed at representing a typical time to schedule an appointment, accommodating for variation in practice. The approach was outlined with the input from local foot specialists
- *Overall Care Scores*. We used the number of visits corresponding to criteria ii and iii combined and their respective waiting time (v), to stratify patients according to two categories with three levels: foot care quality after DFU (low, standard, and high) and cardiovascular-care quality after DFU (low, standard, and high). A high level of care implied at least one or more visits with a waiting time below 12 weeks; a low level of care implied no visits; all other combinations were classified as standard care. These were defined as quality scores considering the dimensions of integration, timeliness, and efficiency (e.g., specialties type, waiting time, and frequency), following the World Health Organization's guidance [30], and considering their alignment with the intended purpose of use as quality indicators [31].

### 2.5. Statistical Analysis

Descriptive analysis included the mean and standard deviation for continuous variables and absolute and relative frequencies for categorical variables. Survival analysis was used to investigate outcomes by taking into account all potential predictors, using censored observations. For LEA events, we defined time-to-event as the difference between the date of first major amputation and the date of DFU diagnosis. For fatal events, we defined time-to-event as the difference between the date of death and the date of DFU diagnosis. For event-free survival, we used as censoring the lag between the date of DFU diagnosis and the date of study closure (31^st^ December 2017).

Different models were separately fit for subjects aged up to 70 and above. In this way, we avoided the potential bias introduced by competing risk for cases deceased before undergoing a major amputation.

Cox proportional hazards was used to calculate univariate and multivariate hazard ratios in time-to-event models. An alpha level of 0.05 was used to report hazard ratios (HRs), their 95% confidence intervals, and *p* values. Forest plots were used to visualize results.

Predictive factors were selected for all multivariate regressions, using a fully automated four-step backward elimination process, with age and gender forced in all models. All other variables were sequentially excluded in three consecutive rounds, using a *p* value ≥ 0.20, ≥0.10, and ≥0.05. General measurable collinearity was tested with variance inflation factors.

All statistical analyses were carried out by developing ad hoc software in the R language [32].

## 3. Results

### 3.1. Descriptive and Univariate Analysis

A total of 6,845 subjects (2.13%) experienced a DFU between 2013 and 2017 out of 321,671 people recorded with type 2 diabetes in Scotland. The median age was of 69.6 years (IQR = 59‐78), and the majority were males (62%). The median duration of follow-up was 79.1 weeks from DFU diagnosis (IQR = 31‐149). The most frequent comorbidities were hypertension (71.6%), retinopathy (41.5%), history of AMI (11.2%), and peripheral vascular disease (10.9%). The variables found most frequently abnormal were eGFR ≤ 59 ml/min (30.1%) and total cholesterol > 5 mmol/L (23%). The process measures with higher adherence were five foot-checks (80.7%) and all the nine care processes in the six months prior to DFU (55.5%). The mean time from DFU diagnosis to the first appointment with a specialist was of 5.7 weeks. Relative frequencies did not differ among target age groups.

In the reference population, 199 subjects (2.9%) reported a major LEA following a diagnosis of DFU during the study period. The results of univariate analyses using LEA as an outcome, for all age groups and stratified up to 70 years and above, are shown in Table 1. A higher proportion of LEA was found among those aged ≤70 years (3.7% vs. 2.0%). An additional year of age was significantly associated with a 2% decreased risk of major LEA (HR = 0.98; 0.97-0.99; *p* < .01). The risk was over four times increased for high foot care quality after DFU (4.74; 3.33-6.77; *p* < .001), foot-checks after DFU (4.40; 2.64-7.35; *p* < .001), minor LEA after DFU (HR = 4.68; 3.35-6.53; *p* < .001), and peripheral vascular disease (HR = 4.05; 3.02-5.43; *p* < .001). Among patients > 70 years, protective factors included foot care visits before DFU (HR = 0.58; 0.3-1.11; *p* = .079) and waiting time > 12 weeks (HR = 0.38; 0.18-0.84; *p* < .01).

All univariate analyses using amputation-free survival (LEA/death) as an outcome, for all age groups and stratified up to 70 years and above, are shown in Table 2.

In the reference population, 2,243 subjects (32.7%) either died or suffered a major LEA in the specified timeframe, with higher rates reported among those aged >70 years (*N* = 1,582; 47.2%), as compared to the younger subgroup (*N* = 661; 18.9%). Moderate increased risk was found for peripheral vascular disease, hypertension, and claudication. The risk of LEA or death was decreased for the following: BMI < 25 (HR = 0.59; 0.53-0.65; *p* < .001), receiving all the nine care processes prior to DFU (HR = 0.66; 0.61-0.72; *p* < .001), foot care visits before DFU (HR = 0.88; 0.8-0.97; *p* < .01), cardiovascular visits after DFU (HR = 0.79; 0.73-0.87; *p* < .001), and total cholesterol > 5 mmol/L (HR = 0.82; 0.73-0.91; *p* < .001). Among those aged ≤70 years, the following characteristics were at increased risk of LEA or death: smoking (HR = 1.7; 1.44-2.01; *p* < .001), deprivation (HR = 1.23; 1.05-1.44; *p* < .01), and minor LEA (HR = 1.31; 1.01-1.69; *p* = .046), as opposed to a waiting time > 12 weeks (HR = 0.71; 0.58-0.87; *p* < .001). Among those aged >70 years, we found a moderately increased risk for smoking (HR = 1.21; 1.04-1.42; *p* = .019) and retinopathy (HR = 1.13; 1.02-1.25; *p* = .02).

### 3.2. Multivariate Analysis

**Figure 1:**
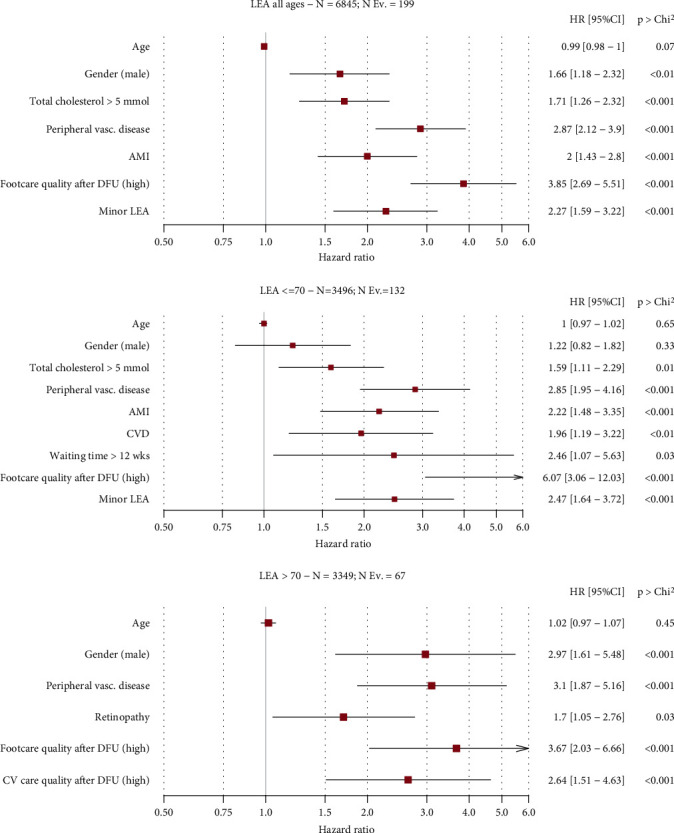
Multivariate analysis for LEA as an outcome.

The results of multivariate analysis using LEA as an outcome are shown in Figure 1.

Among demographic characteristics, only gender (male) was associated with a higher risk of LEA in all age groups (HR = 1.66; 1.18-2.32; *p* = .01), with higher impact among those aged >70 years (HR = 2.97; 1.61-5.48; *p* < .001). Clinical characteristics associated with an increased risk of LEA in all age groups included peripheral vascular disease (HR = 2.87; 2.12-3.9; *p* < .001), AMI (HR = 2; 1.43-2.8; *p* < .001), minor LEA (HR = 2.27; 1.59-3.22; *p* < .001), and total cholesterol > 5 mmol/L (HR = 1.71; 1.26-2.32; *p* < .001). CVD was associated with a higher risk among those aged ≤70 years (HR = 1.96; 1.19-3.22; *p* = .01), while retinopathy was at increased risk among those >70 (HR = 1.7; 1.05-2.76; *p* = .03). Among process measures, a higher risk of LEA was noted for high foot care quality after DFU at all ages (HR = 3.85; 2.69-5.51; *p* < .001), with a higher risk among those ≤70 years (HR = 6.07; 3.06-12.03; *p* < .001). Among patients aged ≤70 years, we found an increased risk of LEA for a waiting time > 12 weeks (HR = 2.46; 1.07-5.63; *p* = .03), while in those >70 years, high cardiovascular-care quality after DFU (HR = 2.64; 1.51-4.63; *p* < .001) was also associated to LEA.

**Figure 2:**
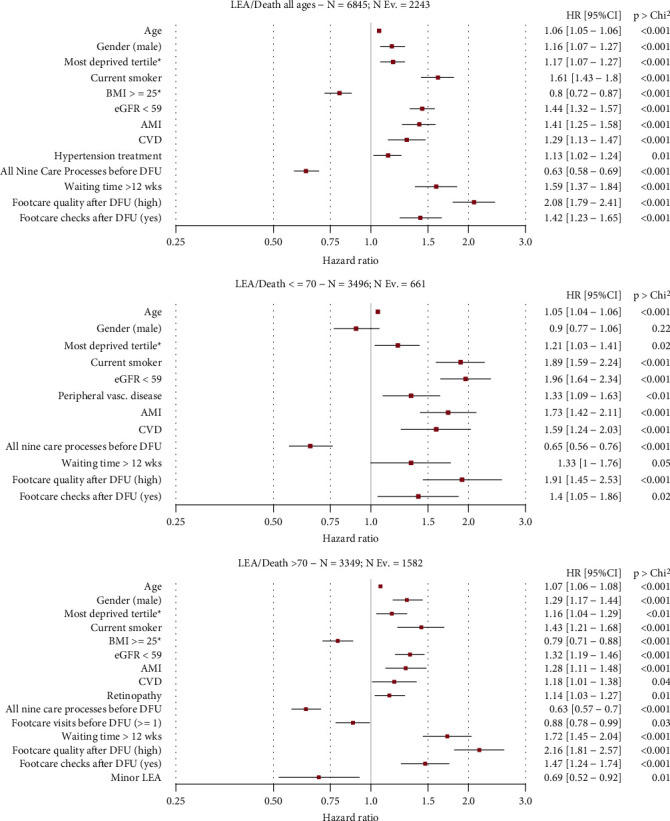
Multivariate analysis for amputation-free survival (LEA/death).

The results of multivariate amputation-free survival (LEA/death) analysis are shown in Figure 2.

Among sociodemographic and lifestyle characteristics, age (HR = 1.06; 1.05-1.06; *p* < .001), male gender (HR = 1.16; 1.07-1.27; *p* < .001), higher deprivation (HR = 1.17; 1.07-1.27; *p* < .001), and smoking (HR = 1.61; 1.43-1.18; *p* < .001) were associated with a higher risk of death or LEA in all age groups, as opposed to a BMI ≥ 25 kg/m^2^, which was found to be protective (HR = 0.8; 0.72-0.87; *p* < .001). Clinical characteristics associated with an increased risk of death or LEA in all age groups were as follows: eGFR ≤ 59 ml/min (HR = 1.44; 1.32-1.57; *p* < .001), AMI (HR = 1.41; 1.25-1.58; *p* < .001), CVD (HR = 1.29; 1.13-1.47; *p* < .001), and hypertension (HR = 1.13; 1.02-1.24; *p* = .001). Among those aged >70 years, a higher risk was also found for retinopathy (HR = 1.14; 1.03-1.27; *p* = .01), while minor LEA (HR = 0.69; 0.52-0.92; *p* = .01) and BMI ≥ 25 (HR = 0.79; 0.71-0.88) were associated to a lower risk. Concerning process measures, protective factors included receiving all nine care processes prior to DFU at all age groups (HR = 0.63; 0.58-0.69; *p* < .001) and foot care visits before DFU among those aged >70 (HR = 0.88; 0.78-0.99; *p* = .03). Risk factors of LEA and death across the whole population included age and waiting time > 12 weeks (HR = 1.59; 1.37-1.84; *p* < .001), high foot care quality after DFU (2.08; 1.79-2.41; *p* < .001), and foot care checks after DFU (HR = 1.42; 1.23-1.65; *p* < .001). The risk associated to waiting time > 12 weeks was higher in those >70 years (HR = 1.72; 1.45-2.04; *p* < .001) than in the younger subgroup. In contrast, the lower risk associated to all nine care processes prior to DFU was similar between age groups.

Results for general measurable collinearity for both models were below the threshold for collinearity (<10).

## 4. Discussion

Routinely recorded data from the SCI-DC registry was successfully extracted to obtain the following key findings.

Firstly, targeted process measures were associated with improved major amputation-free survival. They include receiving all nine care processes during the six months prior to the DFU regardless of age (HR = 0.63; 0.58-0.69; *p* < .001), and, for patients > 70 years of age, a higher number of foot care visits before DFU (HR = 0.88; 0.78-0.99; *p* = .03). This shows that timely involvement of patients in routine primary/foot care before foot ulceration arises may in fact predict outcomes following diagnosis. To the best of our knowledge, these endpoints have not been previously reported, although they are consistent with similar reports where primary care processes are associated to better outcomes [7, 8]. A practical implication is that not having recently completed the nine care processes before a diagnosis of DFU provides clinicians with an immediate flag associated with poor outcomes, which can be directly assessed using electronic health records. As a clinical indicator, it can trigger alerts for specialist assistance, well before the occurrence of complications. Further research is needed to highlight which type of care would benefit patients with a low involvement in primary care prior to their first DFU.

Secondly, patients with a DFU who wait longer for outpatient care provided by a podiatrist, vascular or orthopaedic specialist, are exposed to 50% combined increased risk of LEA or death, while those aged ≤70 years are additionally exposed to a 2.5 times higher risk of undergoing a major amputation. This highlights the importance of a prompt evaluation of patients by foot specialists, immediately following DFU diagnosis. It may also highlight potential disparities in the timely access to health services across populations, similarly to what has been reported for other complications in diabetes [33, 34]. A practical implication of this finding is that healthcare planning should focus on developing pathways of care to reduce waiting times and fast-track patients diagnosed with foot ulcer to key specialists. Future research should identify the hindering factors that impair timely access to these services, e.g., deprivation, sociodemographic, and health system characteristics.

Thirdly, patients undergoing a minor LEA after DFU show an increased risk of a subsequent major LEA when aged ≤70 years, but a longer amputation-free survival when aged >70 years. While previous studies also report high reamputation rates in patients undergoing LEAs [35], we found that there are differences in reamputation risk influenced by the type of LEA (minor vs. major) and the age of the patient. Our results show that minor LEAs were associated with improved outcomes among older patients. This may be due to accelerated wound healing times, reducing sepsis-related risk of death. A practical implication around this finding is that through the methodology employed in this study, we showed how routine data from electronic health records could be used to monitor the incident cases of minor and major LEAs in longitudinal cohorts. Given the rise in minor LEAs and decrease in major LEAs found in OECD countries [19, 36], further research is needed to investigate the relation between LEA types and their long term outcomes, the interpretation of international trends in terms of quality of care, and the subgroups of patients that can best benefit from different treatments.

Fourthly, deprivation was associated with 17% increased risk of major LEA or death. This adds to previous reports in the literature showing that the most deprived segments are associated with poorer outcomes of foot care in diabetes [37, 38]. Further research is needed to disentangle the individual component of deprivation from service-related characteristics at population level, e.g., ambulatory care provided by services located in deprived areas [39]. Such information is paramount to address deprivation by strengthening targeted actions, e.g., reducing waiting times to specialist foot care after DFU and/or improving patient engagement in the use of currently accessible community services [37, 39].

As a final point, other independent risk factors were found to be associated with major LEA or death, consistently with the relevant literature [8]. High-risk factors include male gender, smoking, peripheral vascular disease, AMI, CVD, hypertension, and elevated total cholesterol. The results highlight the importance of high-risk comorbidities, confirming the validity of our approach. Similarly, we found that BMI ≥ 25 is protective against major LEA or death among patients over 70. Although apparently counterintuitive, the result confirms the higher risks of a low BMI in elderly patients, when it can be associated with higher risks of sarcopenia, frailty, and death [40].

A high quality of care provided after DFU was also at increased risk. The result should be taken as an indicator of the targeted profile of care assigned by a highly standardised service, e.g., the one provided to people with diabetes in Scotland, where diabetes care is stratified according to an agreed set of clinical guidelines. Therefore, it should not surprise that patients in need of more services do actually use the National Health Service increasingly, until they experience the most severe complications.

### 4.1. Limitations and Strengths

The study population was limited by the inclusion of DFU recording as a strict requirement, leading to LEA rates that were reportedly lower than those of other studies [37]. However, this allowed focusing on a population that can be clearly defined as a clinical target of primary care services. The practical utility lies in its direct eligibility for assigned pathways at different levels of primary and secondary care.

The ethnic composition of the sample was homogenous, limiting the generalisation of our findings. However, given the existence of a national registry, the sample is clearly representative of the Scottish population. Care processes are consistent with those adopted in European guidelines; hence, our findings can be compared to a large group of international healthcare systems with similar structures and processes. Similarly, the characterisation of deprivation within the population did not explicitly stratify persons living in nursing homes, which may constitute a significant part of the population with DFUs [41].

Finally, the retrospective observational study design does not allow interpreting our associations in terms of causes and effects. However, our methodology can be reliably applied to other large-scale national registries, enabling international comparisons of the same type of outcomes. This can enhance the reproducibility of our results, while orienting further research, where causality can be explored.

### 4.2. Conclusions

Strict adherence to a standardised package of general diabetes care before foot ulceration and timely foot care visits after ulceration can significantly reduce LEA and death in people with type 2 diabetes. The result calls on clinicians and health systems to target and engage high-risk patients at an early stage. Further research is needed to design pathways of specialist care for those not receiving adequate assistance prior to foot ulceration.

Electronic health records should be considered, whenever available, to assess the level of adherence to optimal care packages, as it can be used to identify high-risk patients. The approach here presented can be adopted by diabetes registries and data sources from different health systems [42, 43] to extract comparable longitudinal cohorts and safely share essential information [44–46] to compare care packages under varying conditions.

## Figures and Tables

**Table 1:** Frequencies and univariate hazard ratios for major LEA as outcome in people with type 2 diabetes and DFU in Scotland.

Variable	Category	All ages				≤70				>70			
		No LEA	LEA	Univariate HR (95% CI)	*p* > Chi	No LEA	LEA	Univariate HR (95% CI)	*p* > Chi	No LEA	LEA	Univariate HR (95%CI)	*p* > Chi
Age	Continuous	68.31 (12.74)	64.44 (12)	0.98 (0.97-0.99)	<0.01	58.07 (8.78)	57.68 (8.07)	1.00 (0.98-1.02)	0.786	78.81 (5.64)	77.75 (5.61)	0.98 (0.93-1.02)	0.288

Gender	Female (R.C.)	2556 (38.5)	46 (23.1)	1.00 (-)		1064 (31.6)	33 (25.0)	1.00 (-)		1492 (45.5)	13 (19.4)	1.00 (-)	
	Male	4090 (61.5)	153 (76.9)	2.06 (1.48-2.86)	<0.001	2300 (68.4)	99 (75.0)	1.38 (0.93-2.05)	0.099	1790 (54.5)	54 (80.6)	3.57 (1.95-6.54)	<0.001

SIMD score	≥4 (R.C.)	3728 (56.1)	101 (50.8)	1.00 (-)		1713 (50.9)	58 (43.9)	1.00 (-)		2015 (61.4)	43 (64.2)	1.00 (-)	
	<3	2628 (39.5)	88 (44.2)	1.26 (0.94-1.67)	0.118	1498 (44.5)	70 (53.0)	1.43 (1.01-2.03)	0.043	1130 (34.4)	18 (26.9)	0.77 (0.44-1.34)	0.344
	Missing	290 (4.4)	10 (5.0)	1.24 (0.65-2.37)	0.534	153 (4.5)	4 (3.0)	0.74 (0.27-2.04)	0.544	137 (4.2)	6 (9.0)	2.04 (0.87-4.79)	0.135

Smoker	Past/never (R.C.)	5580 (84.0)	155 (77.9)	1.00 (-)		2625 (78.0)	94 (71.2)	1.00 (-)		2955 (90.0)	61 (91.0)	1.00 (-)	
	Current	1066 (16.0)	44 (22.1)	1.53 (1.09-2.14)	0.017	739 (22.0)	38 (28.8)	1.53 (1.05-2.23)	0.033	327 (10.0)	6 (9.0)	0.95 (0.41-2.19)	0.899

BMI	≥25 (R.C.)	1133 (17.0)	28 (14.1)	1.00 (-)		353 (10.5)	20 (15.2)	1.00 (-)		780 (23.8)	8 (11.9)	1.00 (-)	
	<25	5144 (77.4)	160 (80.4)	1.18 (0.79-1.76)	0.416	2766 (82.2)	106 (80.3)	0.71 (0.44-1.14)	0.175	2378 (72.5)	54 (80.6)	2.03 (0.97-4.26)	0.042
	Missing	369 (5.6)	11 (5.5)	1.13 (0.56-2.26)	0.742	245 (7.3)	6 (4.5)	0.44 (0.18-1.10)	0.061	124 (3.8)	5 (7.5)	4.04 (1.32-12.39)	0.024

eGFR	>59 (R.C.)	3979 (59.9)	132 (66.3)	1.00 (-)		2446 (72.7)	97 (73.5)	1.00 (-)		1533 (46.7)	35 (52.2)	1.00 (-)	
	≤59	2003 (30.1)	58 (29.1)	0.99 (0.73-1.35)	0.945	551 (16.4)	29 (22.0)	1.42 (0.94-2.15)	0.109	1452 (44.2)	29 (43.3)	0.96 (0.59-1.57)	0.874
	Missing	664 (10.0)	9 (4.5)	0.41 (0.21-0.80)	<0.01	367 (10.9)	6 (4.5)	0.40 (0.17-0.90)	0.011	297 (9.0)	3 (4.5)	0.45 (0.14-1.47)	0.138

Total cholesterol	≤5 mmol (R.C.)	5093 (76.6)	130 (65.3)	1.00 (-)		2373 (70.5)	77 (58.3)	1.00 (-)		2720 (82.9)	53 (79.1)	1.00 (-)	
	>5 mmol	1507 (22.7)	69 (34.7)	1.80 (1.34-2.40)	<0.001	968 (28.8)	55 (41.7)	1.78 (1.26-2.52)	<0.01	539 (16.4)	14 (20.9)	1.39 (0.77-2.51)	0.286
	Missing	46 (0.7)	0 (0.0)	0.00 (0.00-Inf)	0.168	23 (0.7)	0 (0.0)	0.00 (0.00-Inf)	0.283	23 (0.7)	0 (0.0)	0.00 (0.00-Inf)	0.384

Peripheral vasc. disease	No (R.C.)	5968 (89.8)	132 (66.3)	1.00 (-)		3039 (90.3)	89 (67.4)	1.00 (-)		2929 (89.2)	43 (64.2)	1.00 (-)	
	Yes	678 (10.2)	67 (33.7)	4.05 (3.02-5.43)	<0.001	325 (9.7)	43 (32.6)	4.16 (2.89-5.98)	<0.001	353 (10.8)	24 (35.8)	4.12 (2.50-6.78)	<0.001

Claudication	No	5843 (87.9)	176 (88.4)	1.00 (-)		2938 (87.3)	116 (87.9)	1.00 (-)		2905 (88.5)	60 (89.6)	1.00 (-)	
	Yes	291 (4.4)	17 (8.5)	2.00 (1.22-3.30)	0.013	156 (4.6)	12 (9.1)	2.09 (1.15-3.79)	0.028	135 (4.1)	5 (7.5)	1.76 (0.71-4.38)	0.262
	Missing	512 (7.7)	6 (3.0)	0.41 (0.18-0.92)	0.013	270 (8.0)	4 (3.0)	0.39 (0.14-1.05)	0.03	242 (7.4)	2 (3.0)	0.43 (0.10-1.74)	0.172

AMI	No (R.C.)	5925 (89.2)	154 (77.4)	1.00 (-)		3028 (90.0)	100 (75.8)	1.00 (-)		2897 (88.3)	54 (80.6)	1.00 (-)	
	Yes	721 (10.8)	45 (22.6)	2.49 (1.79-3.48)	<0.001	336 (10.0)	32 (24.2)	2.88 (1.93-4.29)	<0.001	385 (11.7)	13 (19.4)	1.92 (1.05-3.52)	0.049

CVD	No (R.C.)	6099 (91.8)	174 (87.4)	1.00 (-)		3145 (93.5)	113 (85.6)	1.00 (-)		2954 (90.0)	61 (91.0)	1.00 (-)	
	Yes	547 (8.2)	25 (12.6)	1.68 (1.11-2.56)	0.023	219 (6.5)	19 (14.4)	2.55 (1.57-4.15)	<0.001	328 (10.0)	6 (9.0)	0.91 (0.39-2.10)	0.815

Hypertension treatment	No (R.C.)	1828 (27.5)	42 (21.1)	1.00 (-)		970 (28.8)	28 (21.2)	1.00 (-)		858 (26.1)	14 (20.9)	1.00 (-)	
	Yes	4747 (71.4)	157 (78.9)	1.47 (1.05-2.07)	0.021	2345 (69.7)	104 (78.8)	1.50 (0.99-2.27)	0.049	2402 (73.2)	53 (79.1)	1.44 (0.80-2.59)	0.214
	Missing	71 (1.1)	0 (0.0)	0.00 (0.00-Inf)	0.097	49 (1.5)	0 (0.0)	0.00 (0.00-Inf)	0.124	22 (0.7)	0 (0.0)	0.00 (0.00-Inf)	0.436

Retinopathy	No (R.C.)	3737 (56.2)	84 (42.2)	1.00 (-)		1661 (49.4)	53 (40.2)	1.00 (-)		2076 (63.3)	31 (46.3)	1.00 (-)	
	Yes	2729 (41.1)	111 (55.8)	1.73 (1.30-2.29)	<0.001	1603 (47.7)	76 (57.6)	1.43 (1.01-2.03)	0.044	1126 (34.3)	35 (52.2)	2.05 (1.26-3.32)	<0.01
	Missing	180 (2.7)	4 (2.0)	1.15 (0.42-3.13)	0.791	100 (3.0)	3 (2.3)	1.07 (0.33-3.41)	0.914	80 (2.4)	1 (1.5)	1.02 (0.14-7.50)	0.982

Nine care processes before DFU	<9 (R.C.)	2957 (44.5)	88 (44.2)	1.00 (-)		1451 (43.1)	63 (47.7)	1.00 (-)		1506 (45.9)	25 (37.3)	1.00 (-)	
	9	3689 (55.5)	111 (55.8)	0.82 (0.62-1.09)	0.169	1913 (56.9)	69 (52.3)	0.72 (0.51-1.01)	0.056	1776 (54.1)	42 (62.7)	1.10 (0.67-1.81)	0.699

Foot care visits before DFU	No (R.C.)	4964 (74.7)	148 (74.4)	1.00 (-)		2493 (74.1)	92 (69.7)	1.00 (-)		2471 (75.3)	56 (83.6)	1.00 (-)	
	Yes	1682 (25.3)	51 (25.6)	1.02 (0.74-1.40)	0.918	871 (25.9)	40 (30.3)	1.29 (0.89-1.87)	0.184	811 (24.7)	11 (16.4)	0.58 (0.30-1.11)	0.079

Waiting time > 12 wks	≤12 wks (R.C.)	5064 (76.2)	177 (88.9)	1.00 (-)		2583 (76.8)	117 (88.6)	1.00 (-)		2481 (75.6)	60 (89.6)	1.00 (-)	
	>12wks	1582 (23.8)	22 (11.1)	0.42 (0.27-0.66)	<0.001	781 (23.2)	15 (11.4)	0.44 (0.26-0.76)	<0.01	801 (24.4)	7 (10.4)	0.38 (0.18-0.84)	<0.01

Foot care quality after DFU	Low	3008 (45.3)	38 (19.1)	1.00 (-)		1508 (44.8)	24 (18.2)	1.00 (-)		1500 (45.7)	14 (20.9)	1.00 (-)	
	High	3638 (54.7)	161 (80.9)	4.74 (3.33-6.77)	<0.001	1856 (55.2)	108 (81.8)	4.71 (3.02-7.34)	<0.001	1782 (54.3)	53 (79.1)	4.53 (2.51-8.20)	<0.001

Foot care checks after DFU	No (R.C.)	1307 (19.7)	16 (8.0)	1.00 (-)		667 (19.8)	9 (6.8)	1.00 (-)		640 (19.5)	7 (10.4)	1.00 (-)	
	Yes	5339 (80.3)	183 (92.0)	4.40 (2.64-7.35)	<0.001	2697 (80.2)	123 (93.2)	5.08 (2.58-10.01)	<0.001	2642 (80.5)	60 (89.6)	3.38 (1.54-7.43)	<0.001

CV visits after DFU	0 (R.C.)	4296 (64.6)	101 (50.8)	1.00 (-)		1858 (55.2)	62 (47.0)	1.00 (-)		2438 (74.3)	39 (58.2)	1.00 (-)	
	≥1	2350 (35.4)	98 (49.2)	1.67 (1.27-2.21)	<0.001	1506 (44.8)	70 (53.0)	1.35 (0.96-1.89)	0.088	844 (25.7)	28 (41.8)	2.02 (1.24-3.28)	<0.01

CV care quality after DFU	Low (R.C.)	5688 (85.6)	156 (78.4)	1.00 (-)		2709 (80.5)	106 (80.3)	1.00 (-)		2979 (90.8)	50 (74.6)	1.00 (-)	
	High	958 (14.4)	43 (21.6)	1.63 (1.16-2.28)	<0.01	655 (19.5)	26 (19.7)	1.05 (0.68-1.61)	0.825	303 (9.2)	17 (25.4)	3.32 (1.92-5.77)	<0.001

Minor LEA	No (R.C.)	6371 (95.9)	154 (77.4)	1.00 (-)		3180 (94.5)	97 (73.5)	1.00 (-)		3191 (97.2)	57 (85.1)	1.00 (-)	
	Yes	275 (4.1)	45 (22.6)	4.68 (3.35-6.53)	<0.001	184 (5.5)	35 (26.5)	4.44 (3.01-6.54)	<0.001	91 (2.8)	10 (14.9)	4.33 (2.21-8.50)	<0.001

**Table 2:** Frequencies and univariate hazard ratios for amputation-free survival (LEA/death) in people with type 2 diabetes and DFU in Scotland.

Variable	Category	All ages				≤70				>70			
		No LEA	LEA	Univariate HR (95% CI)	*p* > Chi	No LEA	LEA	Univariate HR (95% CI)	*p* > Chi	No LEA	LEA	Univariate HR (95% CI)	*p* > Chi
Age	Continuous	64.99 (12.34)	74.76 (10.86)	1.06 (1.05-1.06)	<0.001	57.32 (8.87)	61.2 (7.49)	1.05 (1.04-1.07)	<0.001	77.31 (4.94)	80.43 (5.91)	1.07 (1.06-1.07)	<0.001

Gender	Female (R.C.)	1689 (36.7)	913 (40.7)	1.00 (-)		871 (30.7)	226 (34.2)	1.00 (-)		818 (46.3)	687 (43.4)	1.00 (-)	
	Male	2913 (63.3)	1330 (59.3)	0.90 (0.83-0.98)	0.015	1964 (69.3)	435 (65.8)	0.88 (0.75-1.04)	0.139	949 (53.7)	895 (56.6)	1.12 (1.02-1.24)	0.023

SIMD score	≥4 (R.C.)	2560 (55.6)	1269 (56.6)	1.00 (-)		1462 (51.6)	309 (46.7)	1.00 (-)		1098 (62.1)	960 (60.7)	1.00 (-)	
	<3	1848 (40.2)	868 (38.7)	0.98 (0.90-1.07)	0.71	1247 (44.0)	321 (48.6)	1.23 (1.05-1.44)	<0.01	601 (34.0)	547 (34.6)	1.05 (0.94-1.16)	0.379
	Missing	194 (4.2)	106 (4.7)	1.03 (0.85-1.26)	0.755	126 (4.4)	31 (4.7)	1.08 (0.74-1.56)	0.702	68 (3.8)	75 (4.7)	1.14 (0.90-1.44)	0.277

Smoker	Past/never (R.C.)	3872 (84.1)	1863 (83.1)	1.00 (-)		2263 (79.8)	456 (69.0)	1.00 (-)		1609 (91.1)	1407 (88.9)	1.00 (-)	
	Current	730 (15.9)	380 (16.9)	1.10 (0.98-1.22)	0.107	572 (20.2)	205 (31.0)	1.70 (1.44-2.01)	<0.001	158 (8.9)	175 (11.1)	1.21 (1.04-1.42)	0.019

BMI	≥25 (R.C.)	611 (13.3)	550 (24.5)	1.00 (-)		278 (9.8)	95 (14.4)	1.00 (-)		333 (18.8)	455 (28.8)	1.00 (-)	
	<25	3717 (80.8)	1587 (70.8)	0.59 (0.53-0.65)	<0.001	2347 (82.8)	525 (79.4)	0.74 (0.59-0.92)	<0.01	1370 (77.5)	1062 (67.1)	0.69 (0.62-0.77)	<0.001
	Missing	274 (6.0)	106 (4.7)	0.55 (0.44-0.67)	<0.001	210 (7.4)	41 (6.2)	0.64 (0.45-0.93)	0.015	64 (3.6)	65 (4.1)	0.89 (0.68-1.15)	0.355

eGFR	>59 (R.C.)	3073 (66.8)	1038 (46.3)	1.00 (-)		2151 (75.9)	392 (59.3)	1.00 (-)		922 (52.2)	646 (40.8)	1.00 (-)	
	≤59	1054 (22.9)	1007 (44.9)	2.20 (2.01-2.40)	<0.001	384 (13.5)	196 (29.7)	2.38 (2.00-2.82)	<0.001	670 (37.9)	811 (51.3)	1.47 (1.32-1.63)	<0.001
	Missing	475 (10.3)	198 (8.8)	1.13 (0.97-1.32)	0.112	300 (10.6)	73 (11.0)	1.18 (0.92-1.52)	0.192	175 (9.9)	125 (7.9)	1.03 (0.85-1.25)	0.766

Total cholesterol	≤5 mmol (R.C.)	3430 (74.5)	1793 (79.9)	1.00 (-)		1968 (69.4)	482 (72.9)	1.00 (-)		1462 (82.7)	1311 (82.9)	1.00 (-)	
	>5 mmol	1143 (24.8)	433 (19.3)	0.82 (0.73-0.91)	<0.001	849 (29.9)	174 (26.3)	0.90 (0.76-1.08)	0.251	294 (16.6)	259 (16.4)	1.05 (0.91-1.19)	0.52
	Missing	29 (0.6)	17 (0.8)	1.30 (0.80-2.09)	0.308	18 (0.6)	5 (0.8)	1.35 (0.56-3.25)	0.528	11 (0.6)	12 (0.8)	1.33 (0.75-2.35)	0.347

Peripheral vasc. disease	No (R.C.)	4164 (90.5)	1936 (86.3)	1.00 (-)		2582 (91.1)	546 (82.6)	1.00 (-)		1582 (89.5)	1390 (87.9)	1.00 (-)	
	Yes	438 (9.5)	307 (13.7)	1.26 (1.12-1.43)	<0.001	253 (8.9)	115 (17.4)	1.81 (1.48-2.21)	<0.001	185 (10.5)	192 (12.1)	1.02 (0.87-1.18)	0.839

Claudication	No	4064 (88.3)	1955 (87.2)	1.00 (-)		2477 (87.4)	577 (87.3)	1.00 (-)		1587 (89.8)	1378 (87.1)	1.00 (-)	
	Yes	210 (4.6)	98 (4.4)	1.05 (0.85-1.28)	0.672	134 (4.7)	34 (5.1)	1.19 (0.84-1.69)	0.331	76 (4.3)	64 (4.0)	1.01 (0.79-1.30)	0.941
	Missing	328 (7.1)	190 (8.5)	1.18 (1.01-1.37)	0.036	224 (7.9)	50 (7.6)	0.99 (0.74-1.32)	0.93	104 (5.9)	140 (8.8)	1.33 (1.11-1.58)	<0.01

AMI	No (R.C.)	4182 (90.9)	1897 (84.6)	1.00 (-)		2588 (91.3)	540 (81.7)	1.00 (-)		1594 (90.2)	1357 (85.8)	1.00 (-)	
	Yes	420 (9.1)	346 (15.4)	1.57 (1.40-1.76)	<0.001	247 (8.7)	121 (18.3)	2.03 (1.66-2.47)	<0.001	173 (9.8)	225 (14.2)	1.35 (1.17-1.55)	<0.001

CVD	No(R.C.)	4281 (93.0)	1992 (88.8)	1.00 (-)		2671 (94.2)	587 (88.8)	1.00 (-)		1610 (91.1)	1405 (88.8)	1.00 (-)	
	Yes	321 (7.0)	251 (11.2)	1.49 (1.31-1.70)	<0.001	164 (5.8)	74 (11.2)	1.93 (1.52-2.46)	<0.001	157 (8.9)	177 (11.2)	1.16 (0.99-1.36)	0.069

Hypertension treatment	No (R.C.)	1330 (28.9)	540 (24.1)	1.00 (-)		844 (29.8)	154 (23.3)	1.00 (-)		486 (27.5)	386 (24.4)	1.00 (-)	
	Yes	3221 (70.0)	1683 (75.0)	1.23 (1.12-1.35)	<0.001	1950 (68.8)	499 (75.5)	1.31 (1.09-1.57)	<0.01	1271 (71.9)	1184 (74.8)	1.17 (1.05-1.32)	<0.01
	Missing	51 (1.1)	20 (0.9)	1.13 (0.72-1.76)	0.602	41 (1.4)	8 (1.2)	1.18 (0.58-2.40)	0.658	10 (0.6)	12 (0.8)	1.64 (0.92-2.92)	0.116

Retinopathy	No (R.C.)	2541 (55.2)	1280 (57.1)	1.00 (-)		1376 (48.5)	338 (51.1)	1.00 (-)		1165 (65.9)	942 (59.5)	1.00 (-)	
	Yes	1961 (42.6)	879 (39.2)	0.90 (0.82-0.98)	0.011	1383 (48.8)	296 (44.8)	0.87 (0.74-1.02)	0.082	578 (32.7)	583 (36.9)	1.13 (1.02-1.25)	0.02
	Missing	100 (2.2)	84 (3.7)	1.62 (1.30-2.02)	<0.001	76 (2.7)	27 (4.1)	1.56 (1.05-2.31)	0.037	24 (1.4)	57 (3.6)	1.98 (1.52-2.59)	<0.001

Nine care processes before DFU	<9 (R.C.)	1940 (42.2)	1105 (49.3)	1.00 (-)		1209 (42.6)	305 (46.1)	1.00 (-)		731 (41.4)	800 (50.6)	1.00 (-)	
	9	2662 (57.8)	1138 (50.7)	0.66 (0.61-0.72)	<0.001	1626 (57.4)	356 (53.9)	0.75 (0.65-0.88)	<0.001	1036 (58.6)	782 (49.4)	0.61 (0.56-0.68)	<0.001

Foot care visits before DFU	No (R.C.)	3386 (73.6)	1726 (77.0)	1.00 (-)		2089 (73.7)	496 (75.0)	1.00 (-)		1297 (73.4)	1230 (77.7)	1.00 (-)	
	Yes	1216 (26.4)	517 (23.0)	0.88 (0.80-0.97)	<0.01	746 (26.3)	165 (25.0)	0.99 (0.83-1.18)	0.88	470 (26.6)	352 (22.3)	0.83 (0.74-0.94)	<0.01

Waiting time > 12 wks	≤12 wks (R.C.)	3477 (75.6)	1764 (78.6)	1.00 (-)		2151 (75.9)	549 (83.1)	1.00 (-)		1326 (75.0)	1215 (76.8)	1.00 (-)	
	>12 wks	1125 (24.4)	479 (21.4)	0.93 (0.84-1.03)	0.171	684 (24.1)	112 (16.9)	0.71 (0.58-0.87)	<0.001	441 (25.0)	367 (23.2)	1.01 (0.90-1.13)	0.885

Foot care quality after DFU	Low	2181 (47.4)	865 (38.6)	1.00 (-)		1310 (46.2)	222 (33.6)	1.00 (-)		871 (49.3)	643 (40.6)	1.00 (-)	
	High	2421 (52.6)	1378 (61.4)	1.82 (1.67-1.98)	<0.001	1525 (53.8)	439 (66.4)	2.11 (1.79-2.48)	<0.001	896 (50.7)	939 (59.4)	1.84 (1.66-2.04)	<0.001

Foot care checks after DFU	No (R.C.)	941 (20.4)	382 (17.0)	1.00 (-)		572 (20.2)	104 (15.7)	1.00 (-)		369 (20.9)	278 (17.6)	1.00 (-)	
	Yes	3661 (79.6)	1861 (83.0)	1.95 (1.75-2.18)	<0.001	2263 (79.8)	557 (84.3)	2.06 (1.67-2.54)	<0.001	1398 (79.1)	1304 (82.4)	2.00 (1.75-2.28)	<0.001

CV visits after DFU	0 (R.C.)	2858 (62.1)	1539 (68.6)	1.00 (-)		1565 (55.2)	355 (53.7)	1.00 (-)		1293 (73.2)	1184 (74.8)	1.00 (-)	
	≥1	1744 (37.9)	704 (31.4)	0.79 (0.73-0.87)	<0.001	1270 (44.8)	306 (46.3)	1.03 (0.89-1.20)	0.682	474 (26.8)	398 (25.2)	0.97 (0.86-1.09)	0.581

CV care quality after DFU	Low (R.C.)	3887 (84.5)	1957 (87.2)	1.00 (-)		2284 (80.6)	531 (80.3)	1.00 (-)		1603 (90.7)	1426 (90.1)	1.00 (-)	
	High	715 (15.5)	286 (12.8)	0.87 (0.77-0.99)	0.026	551 (19.4)	130 (19.7)	1.06 (0.87-1.28)	0.56	164 (9.3)	156 (9.9)	1.10 (0.94-1.30)	0.247

Minor LEA	No (R.C.)	4398 (95.6)	2127 (94.8)	1.00 (-)		2681 (94.6)	596 (90.2)	1.00 (-)		1717 (97.2)	1531 (96.8)	1.00 (-)	
	Yes	204 (4.4)	116 (5.2)	0.85 (0.71-1.03)	0.083	154 (5.4)	65 (9.8)	1.31 (1.01-1.69)	0.046	50 (2.8)	51 (3.2)	0.78 (0.59-1.04)	0.074

## Data Availability

We are unable to make patient level data publicly available because of license restrictions of the Scottish Diabetes Registry. Third parties can request access to the Scottish Diabetes Registry subject to approval.
